# Tenascin-C: Friend or Foe in Lung Aging?

**DOI:** 10.3389/fphys.2021.749776

**Published:** 2021-10-27

**Authors:** Sandrine Gremlich, Tiziana P. Cremona, Eveline Yao, Farah Chabenet, Kleanthis Fytianos, Matthias Roth-Kleiner, Johannes C. Schittny

**Affiliations:** ^1^Clinic of Neonatology, Department Woman-Mother-Child, Lausanne University Hospital and University of Lausanne, Lausanne, Switzerland; ^2^Institute of Anatomy, University of Bern, Bern, Switzerland; ^3^Department for BioMedical Research, University of Bern, Bern, Switzerland; ^4^Division of Pulmonary Medicine, University of Bern, Bern, Switzerland

**Keywords:** lung, extracellular matrix, Tenascin-C, aging, tenascin-C deficiency, lung physiology, stereology

## Abstract

Lung aging is characterized by lung function impairment, ECM remodeling and airspace enlargement. Tenascin-C (TNC) is a large extracellular matrix (ECM) protein with paracrine and autocrine regulatory functions on cell migration, proliferation and differentiation. This matricellular protein is highly expressed during organogenesis and morphogenetic events like injury repair, inflammation or cancer. We previously showed that TNC deficiency affected lung development and pulmonary function, but little is known about its role during pulmonary aging. In order to answer this question, we characterized lung structure and physiology in 18 months old TNC-deficient and wild-type (WT) mice. Mice were mechanically ventilated with a basal and high tidal volume (HTV) ventilation protocol for functional analyses. Additional animals were used for histological, stereological and molecular biological analyses. We observed that old TNC-deficient mice exhibited larger lung volume, parenchymal volume, total airspace volume and septal surface area than WT, but similar mean linear intercept. This was accompanied by an increase in proliferation, but not apoptosis or autophagy markers expression throughout the lung parenchyma. Senescent cells were observed in epithelial cells of the conducting airways and in alveolar macrophages, but equally in both genotypes. Total collagen content was doubled in TNC KO lungs. However, basal and HTV ventilation revealed similar respiratory physiological parameters in both genotypes. Smooth muscle actin (α-SMA) analysis showed a faint increase in α-SMA positive cells in TNC-deficient lungs, but a marked increase in non-proliferative α-SMA + desmin + cells. Major TNC-related molecular pathways were not up- or down-regulated in TNC-deficient lungs as compared to WT; only minor changes in TLR4 and TGFβR3 mRNA expression were observed. In conclusion, TNC-deficient lungs at 18 months of age showed exaggerated features of the normal structural lung aging described to occur in mice between 12 and 18 months of age. Correlated to the increased pulmonary function parameters previously observed in young adult TNC-deficient lungs and described to occur in normal lung aging between 3 and 6 months of age, TNC might be an advantage in lung aging.

## Introduction

In human, lung aging is characterized by a progressive lung function decline, with decreased forced expiratory volume over forced vital capacity ratio (FEV1/FVC), decreased elastic recoil and a “senile emphysema” comparable to chronic obstructive pulmonary disease (COPD) emphysema, but devoid of inflammation and alveolar septa destruction (Brandsma et al., 2017; Navarro and Driscoll, 2017; Bowdish, 2019). Static compliance also raises with age in human (Janssens et al., 1999; Birch et al., 2018). However, in healthy elderly, these functional changes might only be noticeable during exercise (Bowdish, 2019). Remodeling of the parenchyma and airspace enlargement are accompanied by a decrease in elastin content and an increase in collagen content, partly explaining the loss of elastic recoil and the increased stiffness of the lung with age; however, loss of elastic recoil is also a consequence of the reduction in surface tension due to the increased airspace volume (Janssens et al., 1999; Brandsma et al., 2017; Navarro and Driscoll, 2017). Immunosenescence occurring in aging is the result of a decline in the immune system, objectified by a higher susceptibility to infections, auto-immune diseases or cancer and the development of a chronic basal mild inflammatory state called “inflammaging” by Franceschi and colleagues (Franceschi et al., 2000; Brandsma et al., 2017; Bowdish, 2019). Human studies being hard to realize and standardize, more complete studies on lung aging were undertaken in rodents. They ended up with similar findings, especially an increase in static compliance and total inspiratory capacity as well as an increase in lung volume and airspace volume in old mice. However, they allowed a more precise description of structural/microstructural evolution of the lung with age (Yamamoto et al., 2003; Huang et al., 2007; Elliott et al., 2016; Kling et al., 2017; Schulte et al., 2019; Veldhuizen et al., 2019).

Tenascin-C (TNC) is a glycoprotein of the extracellular matrix (ECM) which is highly expressed during organogenesis, but very little or not at all thereafter, except in tissues submitted to high tensile stress or in some stem cell niches. TNC is transiently *de novo* re-expressed in adults during tissue repair following injury or inflammation, or continuously in chronic pathological situations like cancer (Chiquet-Ehrismann and Chiquet, 2003; Chiquet-Ehrismann, 2004; Midwood and Orend, 2009; Chiquet-Ehrismann et al., 2014). As a matricellular protein, TNC binds to cell surface receptors, ECM proteins, soluble factors or pathogens, to modulate cell adhesion, proliferation, migration and differentiation properties in an autocrine and paracrine manner (Chiquet-Ehrismann and Chiquet, 2003; Hsia and Schwarzbauer, 2005; Midwood et al., 2011, 2016). TNC was shown to be an important component of lung development, with an impairment of lung branching morphogenesis (early lung development) and delayed classical and continued alveolarization processes (late lung development) occurring in TNC-deficient mice (Roth-Kleiner et al., 2004; Schittny, 2018; Mund and Schittny, 2020). However, a compensatory mechanism, maybe through other members of the tenascin family resulted in a catch-up of the morphometric measurements of the lung at 3 months of age (Mund and Schittny, 2020). It is likely, that part of the developmental role of TNC is mediated by α8β1 integrin. Indeed, α8β1 integrin recognizes TNC and α8-integrin subunit-deficient mice showed a very similar pulmonary phenotype as TNC-deficient mice (Cremona et al., 2020).

In adult lung, a weak expression of TNC was consistently reported in airway smooth muscle and basal cell layers, while expression in the parenchyma was described as very faint and inconsistent by some authors (Koukoulis et al., 1991; Natali et al., 1991; Kaarteenaho-Wiik et al., 2001; Liesker et al., 2009; Lofdahl et al., 2011). The role of TNC in normal aging was explored essentially in tissues submitted to high tensile stress such as the skin (Choi et al., 2020), cardiac muscle (Sato and Shimada, 2001), ligament/tendon (Fujii et al., 1993; Veronesi et al., 2015; Ribitsch et al., 2020) or even cartilage (Gruber et al., 2002, 2011). In the lung, a single report comparing 24 to 8 months old decellularized mouse lung scaffolds showed that aging was linked to an increase in collagen and collagen-related proteins, and that this increase was preceded by an increase in TNC and osteopontin expression and associated with an increase in the number of senescent cells (Calhoun et al., 2016). A recent review suggested that TNC might, together with other matricellular proteins, contribute to cellular senescence by acting on the cell cycle, apoptosis and the pool of secreted proteins in several tissues among which the lung (Blokland et al., 2020).

General aging hallmarks were proposed to be nine: genomic instability, telomere attrition, epigenetic alterations, loss of proteostasis, deregulated nutrient sensing, mitochondrial dysfunction, cellular senescence, stem cell exhaustion and altered intercellular communication (Lopez-Otin et al., 2013). More recently, Meiners et al. (2015) proposed a tenth hallmark of aging specific for lung aging, which was “dysregulation of the extracellular matrix”. Major chronic lung diseases are exacerbated by aging hallmarks, which put elderly people at higher risk of developing chronic lung diseases such as idiopathic pulmonary fibrosis (IPF), COPD or lung cancer (Meiners et al., 2015; Brandsma et al., 2017; Navarro and Driscoll, 2017). TNC is highly expressed in such chronic lung diseases as COPD, bronchopulmonary dysplasia (BPD), respiratory distress syndrome (RDS), IPF or asthma (Laitinen et al., 1997; Kaarteenaho-Wiik et al., 2002; Lofdahl et al., 2011; Estany et al., 2014; Yasuda et al., 2018). In this paper, we aimed at deciphering whether TNC could play a role in lung aging. We found that TNC knockout (TNC KO) animals at 18 months of age presented an increased lung volume, parenchymal volume, total airspace volume and septal surface area, compared to wild-type (WT) counterparts, with no change in functional pressure-volume curves. Increased morphometric parameters were accompanied by an increase in cell proliferation, essentially in the parenchyma. Apoptotic cells were not detected and senescence was only observed in bronchial epithelial cells and alveolar macrophages in both genotypes. We conclude that old TNC knockout mice exhibit exaggerated structural hallmarks of lung aging, compared to WT counterparts.

## Materials and Methods

### Animals

All experiments were approved by the animal ethic commissions of the Federal Food Safety and Veterinary Office, and the Veterinary Service of the Canton Bern, and performed in accordance with the Swiss Federal Act in Animal protection guidelines and regulations. TNC KO mice (Forsberg et al., 1996) were bred on a 129SV background. Experiments were done with male and female animals. Results of both sexes were only discussed if differences were detected. If no sex-dependent differences were observed, the results of only one sex is shown. Eighteen months old TNC KO and WT animals were anesthetized with a mixture of Midazolam (5 mg/kg BW), Fentanyl (0.05 mg/kg BW) and Medetomidin (0.5 mg/kg BW), then intratracheally intubated and connected to a computer-controlled piston ventilator (flexiVent, SCIREQ Scientific Respiratory Equipment Inc., Montreal, QC, Canada) for mechanical ventilation and for the construction of pressure-driven pressure-volume (PV) curves. To begin with, animals were ventilated with a standard mouse ventilation profile (respiratory rate: 150/min; tidal volume: 10 ml/kg; PEEP: 3 cm H_2_O) and PV curves constructed in a step-wise manner under closed-chest conditions (basal conditions). Then, mechanical ventilation was switched to a high amplitude ventilation mode (HTV for high tidal volume) and the subjects were ventilated for 1 h with room air at a respiratory rate of 60/min, a tidal volume of 25 ml/kg and a PEEP of 0 cm H_2_O. During that time, PV curves were recorded every 15 min. During the whole procedure, animals were kept on a heating pad with rectal temperature maintained at 35–37°C and heart rate monitored by ECG. The Salazar-Knowles equation (Salazar and Knowles, 1964) was fitted to the pressure and volume signals at each plateau of the deflation limb of the PV curves directly in the flexiVent operating system (flexiWare version 7.2) and the following parameters were automatically obtained after each measurement: static compliance (Cst): describing the distensibility of the respiratory system (lungs and chest walls) at 5 cm H_2_O, parameter A: an estimate of the inspiratory capacity and parameter K: the exponential function capturing the curvature of the PV curves. In addition, the area between the inflation and deflation limbs of the PV curve (hysteresis) was calculated. *N* = 3–4 animals/gender and genotype.

At the end of each experiment, the animal was sacrificed with an overdose of the anesthetic mixture. The right main bronchus was ligated and the right lung lobes snap-frozen for RNA/protein extraction. The left lobe was inflated with freshly prepared paraformaldehyde 4% at a pressure of 20 cm H_2_O, fixed and stored for histological analyses. During fixation, pressure was maintained for at least 2 h at 4°C, to prevent lungs from recoiling (Roth-Kleiner et al., 2005). Non-ventilated age-matched animals were sacrificed and recovered the same way for histological, stereological and molecular biology experiments, to be sure to avoid ventilation-induced effects.

### Histology

Once fixed, the volumes of the lung lobes were first measured by water displacement (Scherle, 1970). Then, the left lung lobe was prepared according to the Cavalieri method (Michel and Cruz-Orive, 1988; Cruz-Orive, 1999). The entire lobe was first cut in several slices of 1.5 mm thickness. Depending on the size of the lobe, 5 to 7 slices are obtained (in our experiment 6). The different sections were then put on a plane surface and embedded in paraffin. This method allows to have on the same slide, a global representation of the whole lung lobe, with different sections across the same lobe, from the most distal to the most proximal end of the lobe. Five μm sections were cut using a MicroM microtome (Thermo Fisher Scientific Inc., Reinach, Switzerland). Hematoxylin-eosin staining was done according to the following procedure: 1 min in hematoxylin, 5 min in running tap water, 20 dips in ascending EtOH solutions from 70 to 100%, 20 s in eosin. Resorcin-fuchsin staining was done according to the following procedure: 30 min in Weigert’s resorcin-fuchsin solution, 20 dips in running tap water, 10 s in acid alcohol, 2 min in tap water, 20 dips in distilled water. Masson trichrome staining was done according to the following procedure: 1 min in hematoxylin, 5 min in running tap water, 10 min in fuchsin-Ponceau solution (1% fuchsin, 1% Ponceau in 1% acetic acid), 20 dips in distilled water, 3 min in 1% phosphomolybdic acid, 1 min in fast green FCF (in 1% acetic acid), rinsing in 1% acetic acid. These three staining were done to provide information on the general morphology, elastin and collagen distribution, respectively. For each animal, six pictures of the different lung sections were evaluated by three different examiners (SG, TPC, JCS) for each staining. Criteria for the evaluation of the differences between genotypes were to be able to sort the pictures by genotype, not knowing to which animal it belonged (presence/absence of staining, localization of the staining, amount/intensity of staining, shape/appearance of the staining). WT: *N* = 3–4 animals/gender and TNC KO: *N* = 5 animals/gender.

### Morphometry

A hundred of serial images were taken by slide, according to a systematic random sampling scheme (Cruz-Orive and Weibel, 1981), with a Leica DM RB light microscope (Glattbrugg, Switzerland) equipped with a motorized Maerzheuser XY stage (Wetzlar, Germany), a JVC 930 3-chip color video camera (Oberwil, Switzerland), and the analysis software (Münster, Germany), to cover the entire slide surface, i.e., the 6 sections of a same lung lobe. Once done, half of the images (one in two) were analyzed with the STEPanizer software dedicated to manual stereological assessment of digital images (Tschanz et al., 2011). Lung volume was measured by water displacement before paraffin embedding (Scherle, 1970). Volume density of the lung parenchyma was estimated by point counting (airspaces and septal tissue), and absolute value calculated as the product of volume density multiplied by lung volume. The surface density of the alveolar septa was estimated by intersection counting, and absolute value was calculated as the product of surface density multiplied by lung volume (Howard and Reed, 2005; Hsia et al., 2010). The length density and absolute length of the free septal edges were estimated by counting the tips of septa in a reference area of a lung section: the principle and applied examples are given in Mund et al. (2008), Schittny et al. (2008), Roth-Kleiner et al. (2014), Tschanz et al. (2014), and Mund and Schittny (2020). WT: *N* = 3–4 animals/gender and TNC KO: *N* = 5 animals/gender.

### Western Blots

Frozen lung lobes were ground to powder with the help of a pre-cooled metal mortar and pestle. Powder was resuspended in RIPA buffer (#89900, Pierce, Thermo Fisher Scientific Inc., Waltham, MA, United States) containing added anti-proteases and anti-phosphatases. Protein lysates were quantified with the BCA protein assay (#23225, Pierce, Thermo Fisher Scientific Inc., Waltham, MA, United States). Different amounts of proteins (20, 40 or 100 μg) were loaded on 7.5 or 12.5% SDS-PAGE gels, depending on the level of expression and the size of the protein detected. Gels were transferred on nitrocellulose membranes and Western blot detection was done as follows: blocking of the membrane 30 min at RT in 1× casein solution (#SP-5020); overnight incubation at 4°C with a primary antibody solution in 1× casein containing added 0.1% Tween 20; 1 h incubation at RT with a secondary antibody solution in 1× casein. Primary antibodies used were: anti-α-SMA (#A2547, 1:250), anti-p62/SQSTM1 (#P0067, 1:2,000) and anti-α-actinin (#A5044, 1:250, Sigma-Aldrich Inc., St. Louis, MI, United States), anti LC3b (#AB48394, 1:1,000) and anti-tenascin-C (#AB108930, 1:1,000, Abcam, Cambridge, United Kingdom), anti-PCNA (#sc7907, 1:1,000, Santa Cruz Biotechnology Inc., Dallas, TX, United States), anti-PARP1 (#9542T, 1:1,000, Cell Signaling Technology, Danvers, MA, United States). Secondary antibodies were IRDye 800CW Donkey Anti-Mouse IgG (H + L) (#926-32212) or IRDye 680RD Donkey (polyclonal) Anti-Rabbit IgG (H + L) (#926-68073, LI-COR Biosciences Inc., Lincoln, NE, United States). Signal was revealed using a LI-COR Odyssey Infrared Imaging System (LI-COR Biosciences Inc., Lincoln, NE, United States), then analyzed and quantified with the free ImageJ software. *N* = 6–7 animals/genotype.

### Immunohistochemistry

Five μm sections were cut using a MicroM microtome (Thermo Fisher Scientific Inc., Reinach, Switzerland). Sections were deparaffinized and endogenous peroxydases blocked by H_2_O_2_ treatment (15 min in 0.3% H_2_O_2_ in methanol). When necessary, heat-induced antigen retrieval procedure was applied to the sections (20 min in 0.01 M sodium citrate pH6 at 95°C), before blocking with pre-immune serum. Sections were then incubated during 1–4 h with primary antibodies in PBS 1×, and incubated with the secondary antibody system: biotinylated secondary antibody plus Vectastain ABC HRP kit (#PK-4000). Staining was revealed using Vector SG HRP substrate kit (#SK-4700, Vector Laboratories, Burlingame, CA, United States). Counterstaining was done with Nuclear Fast Red (#6070-5G, Fluka, Buchs, Switzerland). Primary antibodies were anti-α-SMA (#A2547, Sigma-Aldrich, Darmstadt, Germany), anti-ki67 (#NB600-1252, 1:200, Novus Biologicals, Centennial, CO, United States). For α-SMA antibody, Mouse on Mouse Detection Kit (#BMK-2202, Vector Laboratories, Burlingame, CA, United States) was used, to block endogenous mouse Ig staining due to the use of a mouse primary antibody. In each experiment, control slides were added. Negative control was a slide where the primary antibody incubation step was replaced by an incubation step in the primary antibody diluent alone. Positive control was a slide with lung sections of newborn or young adult lungs with a known positive staining with the same antibody. Pictures of different experiments were evaluated by three different examiners (SG, TPC, JCS) for each immunohistochemical staining. Criteria for the evaluation of differences between genotypes were to be able to sort pictures by genotype not knowing to which animal it belonged (presence/absence of staining, localization of the staining, amount/intensity of staining, shape/appearance of the staining). *N* = 3–5 animals/genotype.

### Senescence

The right middle lung lobe of additional animals was inflated with Tissue-Tek O.C.T. Compound (#4583, Sakura Finetek USA, Inc., Torrance, CA, United States) through the trachea, embedded in Tissue-Tek O.C.T. Compound and frozen in a bath of isopentane pre-cooled in liquid nitrogen. Five μm cryosections were cut using a cryostat (HM525 NX cryostat, Thermo Fisher Scientific, Waltham, MA, United States), and tissue was processed with the Senescence Detection Kit (#K320-250, BioVision Inc., Milpitas, CA, United States), following the manufacturer’s protocol. Briefly, sections were fixed 10 min with the Fixative Solution of the kit at RT, and then incubated overnight at 37°C with the Staining Solution Mix containing 1 mg/ml X-gal. Development of the blue color linked with the beta-galactosidase activity was followed under the microscope. *N* = 3 animals/genotype.

### RT-qPCR

Frozen lung lobes were ground to powder with the help of a pre-cooled metal mortar and pestle. Powder was resuspended in TRIzol reagent and total RNA was extracted according to the manufacturer’s protocol (#15596-026, Thermo Fisher Scientific, Waltham, MA, United States). RNA concentration and purity/quality was measured using a NanoDrop 2000 Spectrophotometer (Thermo Fisher Scientific, Waltham, MA, United States). Two μg of RNA were reverse-transcribed using the PrimeScript 1st strand cDNA Synthesis kit (#6110A, Takara Bio Inc., Kusatsu, Japan). PCR were done on a Corbett Rotor-Gene 6000 apparatus, with the Rotor-Gene SYBR Green PCR kit (QIAGEN, Hilden, Germany), following the manufacturer’s protocol. Program was: 40 cycles of 5 s denaturation at 95°C and 10 s annealing/amplification at 60°C. Primers are listed in Supplementary Table 1. *N* = 6 animals/genotype.

### Apoptosis Kit

Apoptosis was evaluated by detection of cell DNA fragmentation with the help of the TACS XL-Blue Label *In Situ* Apoptosis detection kit (#4828-30-BK, Trevigen Inc., Bio-Techne, Minneapolis, MN, United States). Briefly, 5 μm lung sections were permeabilized and incorporation of labeled nucleotides onto the free 3’ OH ends of DNA fragments was followed using BrdU, terminal deoxynucleotidyl transferase enzyme (TdT) and anti-BrdU antibody, according to the manufacturer’s protocol. *N* = 3–5 animals/genotype.

### Flow Cytometry

The left lung lobe of additional animals was resected and homogenized at a single cell level. Briefly, lung lobe was chopped to tiny pieces with scalpels and scissors. Then they were treated with 10 mL collagenase solution (0.1% Collagenase I, 0.25% Collagenase II in in 4% FBS PBS 1×) for 2 h at 37°C/5%CO_2_. Fully supplemented RPMI-1640 was added to deactivate the collagenase solution and cell suspensions were passed through cell strainers of 100 and then 40 μm (#93100 and 93040, SPL Life Sciences Co., Ltd., Pocheon-si, Gyeonggi, South Korea). Immediately after lung homogenization, cells were counted and stained with the Click-iT^TM^ Plus EdU kit (#C10636, Thermo Fischer Scientific Inc., Waltham, MA, United States). Additionally, lung cells were fixed and permeabilized for intracellular flow cytometry staining. Cells were stained with α-Smooth Muscle Actin-PE (#SPM332, Novus Biologicals, Centennial, CO, United States), Vimentin-Alexa 647 (#ab195878) and Desmin (#ab32362) conjugated with Alexa 488 secondary antibody (#ab150073, Abcam, Cambridge, United Kingdom). Intracellular antibody staining was performed on ice. Antibody dilutions and incubation times were performed according to the manufacturers’ specifications. After staining, cells were washed and analyzed at an LSR-II flow cytometer (BD Biosciences, Allschwil, Switzerland). Single cell gate was set as the stopping gate and at least 30,000 events were acquired per sample. Data were analyzed on FlowJo software (Becton Dickinson, Ashland, OR, United States). A representative gating strategy is shown on Supplementary Figure 1. *N* = 3 animals/genotype.

### Hydroxyproline Assay

Total lung collagen content was estimated using hydroxyproline assay as described previously (Tamo et al., 2018). Frozen middle lung lobes were weighted and homogenized. The homogenate was treated with 10% trichloroacetic acid, hydrolyzed with 6 M HCl (overnight, 110°C) and adjusted to pH 7.0 with NaOH. Oxidation was initiated by incubation with 1 ml of chloramine T-reagent (20 min at RT) and stopped by addition of 1 ml of 3.15 M HClO_4_. After incubation with Ehrlich reagent (p-dimethylaminobenzaldehyde added to methyl cellosolve) for 20 min at 55–65°C, absorbance was measured at 557 nm using Tecan M1000 plate reader (Tecan, AG, Männedorf, Switzerland). A standard curve was generated using known concentrations of hydroxyproline.

### Statistical Analyses

Statistical analyses were done by means of GraphPad Prism software (GraphPad software Inc., La Jolla, CA, United States). For respiratory function parameters and flow cytometry experiments, two-way ANOVA were used. For Western blot or RT-qPCR quantifications, Student’s *t* tests were used. Significance was set at *p* < 0.05.

## Results

### Old TNC KO Mice Have a Higher Lung Volume, Parenchymal Volume, Airspace Volume and Septal Surface Area, but a Similar Mean Linear Intercept as Their Wild-Type Counterparts. Total Collagen Content Is Doubled

**FIGURE 1:**
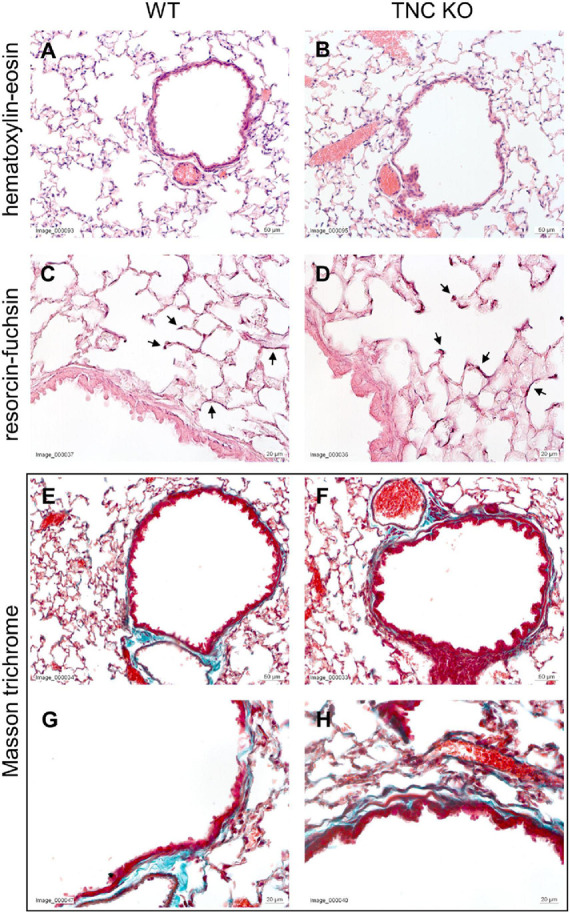
Histological analysis of old TNC-deficient and WT lungs. Representative images of lung sections of female TNC KO and WT lungs at 18 months of age. Five μm lung sections were stained with the following staining; **(A,B)**: hematoxylin-eosin for general morphology; **(C,D)**: resorcin-fuchsin for elastin (dark pink staining; black arrows); **(E–H)**: Masson trichrome for collagen (blue-green staining). WT: *N* = 3–4 animals/gender and TNC KO: *N* = 5 animals/gender.

**FIGURE 2:**
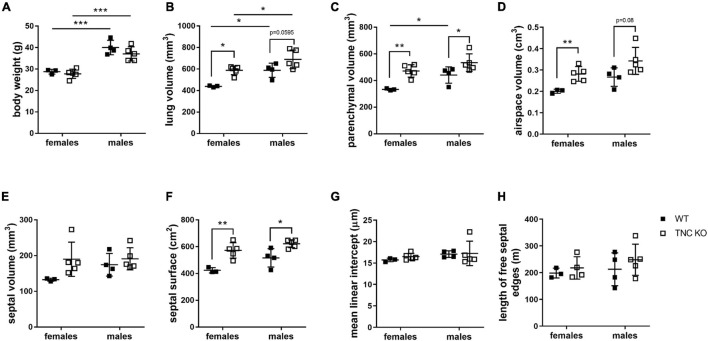
Stereological analysis of old TNC-deficient and WT lungs. Stereological measurements were done on 5 μm sections of 18 months old WT (■) and TNC-deficient (□) females and males lungs. **(A)**: body weight; **(B)**: lung volume; **(C)**: parenchymal volume; **(D)**: airspace volume; **(E)**: septal volume; **(F)**: septal surface area; **(G)**: mean linear intercept; **(H)**: total length of free septal edges. Each point represents one animal; WT: *N* = 3–4 animals/gender and TNC KO: *N* = 5 animals/gender. Results are expressed as mean ± SD. Statistical analysis was made by two-way ANOVA; statistical significance was set at *p* < 0.05; *p < 0.05, **p < 0.01, ***p < 0.001.

To establish the general morphology and morphometry of TNC-deficient and WT lungs at old age (18 months), paraffin blocks containing six slices (same thickness) of the same lung lobe, overall representing the whole lobe (Cavalieri method), were cut and sections stained with hematoxylin-eosin (general morphology), resorcin-fuchsin (elastin staining) and Masson trichrome (collagen staining). Lung morphology of both TNC KO and WT lungs was rather similar at first sight (Figures 1A,B). Elastin and collagen staining did moreover not reveal any evident disparity between TNC KO and WT old lungs (Figures 1C–H). Elastin protein expression was evaluated by Western blot. Its expression was very low, but not obviously different between genotypes (data not shown). However, hydroxyproline assay showed a two-fold increase in total collagen content in TNC KO compared to WT lungs (Supplementary Figure 2). Body weight was found to be higher in old males than in old females, but was not affected by genotype (Figure 2A). Yet, we found a significantly increased lung volume (measured in freshly fixed lungs before embedding), and, stereologically, an increased parenchymal volume, total airspace volume and septal surface area in TNC KO lungs, compared to WT counterparts (Figures 2B–F). All values were increased by 30–40% in the females and 20–30% in the males in TNC KO animals compared to WT animals. Septal volume also tended to be higher in female TNC KO lungs compared to WT lungs (*p* = 0.086), but this was not the case in males (*p* = 0.74). Overall, differences and significance were always more pronounced in females than in males. Mean linear intercept (Lm) was calculated, and showed no significant difference between genotypes, whether in males (*p* = 0.99) or in females (*p* = 0.79) (Figure 2G). The density of the length of the free septal edges, representing the density of the total length of the alveolar entrance rings, also tended to be lower in female (*p* = 0.11) but not in male (*p* = 0.95) TNC KO compared to WT counterparts, but the absolute length of the free septal edges (length density multiplied by parenchymal volume), estimating the total length of the alveolar entrance rings, was similar in old TNC KO and WT lungs, whether female or male (Figure 2H).

### Cell Proliferation Is Increased in the Parenchyma of Old TNC KO Lungs, Compared to Wild-Type Ones

**FIGURE 3:**
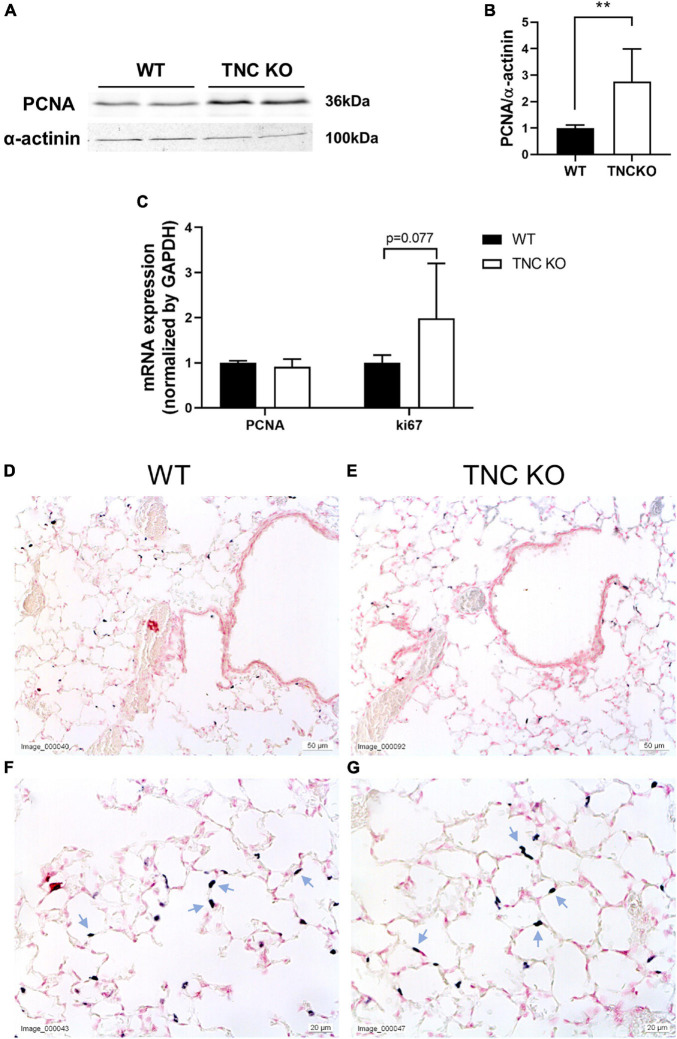
Proliferation markers in old TNC-deficient and WT lungs. **(A,B)**: representative results of PCNA protein detection in whole lung lysates from old TNC-deficient and WT animals by Western blot; quantification is shown in histogram; *N* = 6–7 animals/genotype. Cropped blots are displayed. **(C)**: mRNA expression of PCNA and ki67 evaluated by RT-qPCR from whole lung lobe total RNA extraction of old TNC KO (□) and WT (■) animals. *N* = 6 animals/genotype. **(D–G)**: representative images of lung sections from TNC KO and WT old animals immunostained with anti-ki67 antibody (dark gray; blue arrows) and counter-stained with Nuclear Red. *N* = 3–5 animals/genotype. For histograms: results are expressed as mean ± SD; statistical analyses were made by Student’s *t* test; statistical significance was set at *p* < 0.05; *p < 0.05, **p < 0.01.

**FIGURE 4:**
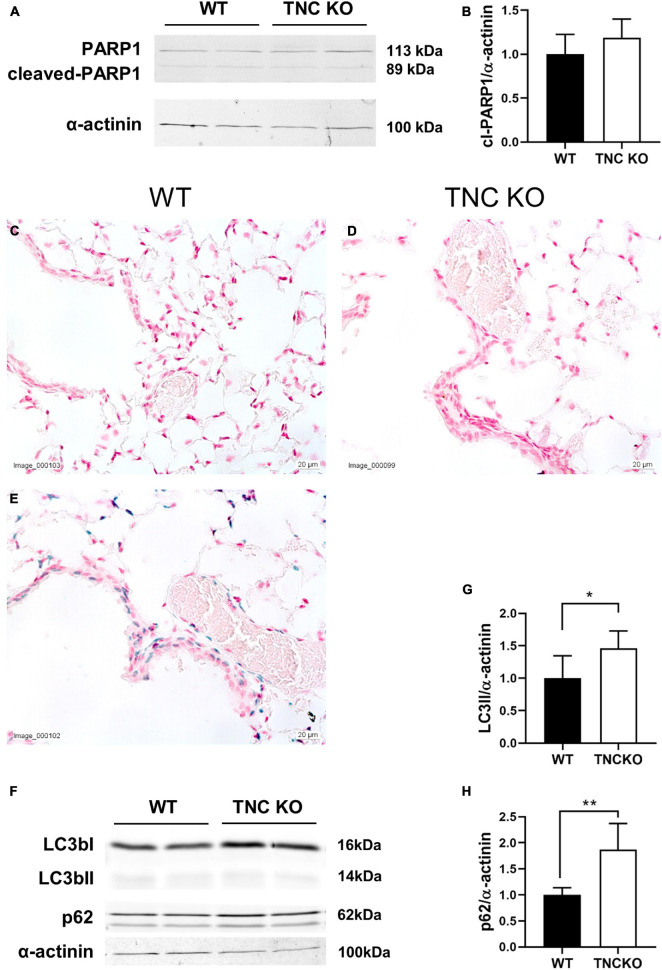
Cell death markers in old TNC-deficient and WT lungs. Evaluation of apoptosis/necrosis and autophagy markers in lungs of TNC-deficient and WT animals. **(A,B)**: representative results of PARP1 protein detection in whole lung lysates from old TNC-deficient and WT animals by Western blot; quantification is shown in histogram; *N* = 6–7 animals/genotype. Cropped blots are displayed. **(C–E)**: representative images of lung sections from TNC KO and WT old animals stained for *in situ* detection of DNA fragmentation typical of apoptosis (blue) and counter-stained with Nuclear Fast Red [**(E)**: positive control]. *N* = 3–5 animals/genotype. **(F–H)**: representative results of LC3b and p62 protein detection in whole lung lysates from old TNC-deficient and WT animals by Western blot; quantification is shown in histogram; *N* = 6–7 animals/genotype. Cropped blots are displayed. For histograms: results are expressed as mean ± SD; statistical analyses were made by Student’s *t* test; statistical significance was set at *p* < 0.05; *p < 0.05, **p < 0.01.

To evaluate if an increased cell proliferation or a decreased cell death rate could be responsible for the increased tissue volume and surface area in TNC-deficient lungs at old age, we measured the expression of several markers of proliferation, apoptosis/necrosis and autophagy in TNC KO and WT lungs (Figures 3, 4). Proliferation markers ki67 (antigen ki-67) and PCNA (proliferating cell nuclear antigen) were assessed by Western blot, RT-qPCR and/or immunohistochemistry. PCNA protein, but not mRNA, expression was almost three-fold higher in TNC KO lungs, compared to WT lungs (Figures 3A–C). Antigen ki67 mRNA expression was two-fold higher in TNC KO lungs than in WT lungs, and immunohistochemical detection of its protein showed that its expression was restricted to the parenchyma (Figures 3D–G). As for autophagy markers, both LC3bII (microtubule-associated protein 1A/1B-light chain 3 beta) and p62/SQSTM1 (sequestosome 1) proteins were found to be higher expressed in TNC KO lungs than in WT lungs (Figures 4A–C). However, activation of autophagy should be evidenced by an increased LC3bII protein but a decreased p62 protein expression. Therefore, autophagy was probably not stimulated in TNC KO lungs. Cleaved PARP1 [poly(ADP-ribose) polymerase 1] and cleaved caspase 3 are both hallmarks of apoptosis and/or necrosis. Cleaved PARP1 protein expression was very low, but similar in both genotypes (Figures 4A,B), while cleaved caspase 3 could simply not be detected by immunohistochemistry (data not shown). *In situ* detection of DNA fragmentation showed that, although the positive control was clearly positive (Figure 4E), no apoptotic cells could be detected either in old TNC KO or WT lung sections, meaning that the level of apoptosis was too low to be detected and apparently not higher in TNC KO lungs, compared to WT ones (Figures 4C,D).

### Senescent Cells Are Observed in Bronchial Epithelial Cells and Alveolar Macrophages in Both Old TNC KO and Wild-Type Mice

**FIGURE 5:**
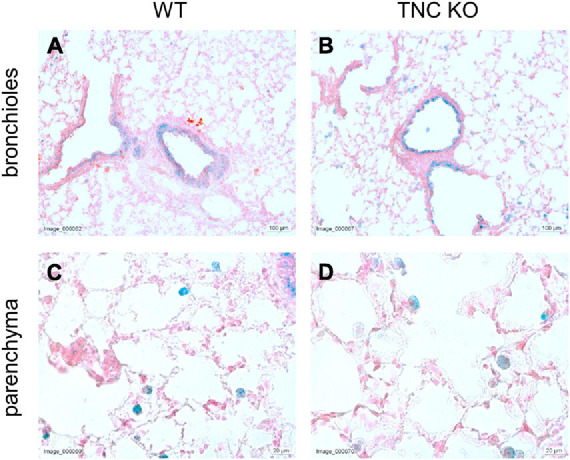
Senescent cells in old TNC-deficient and WT lungs. Senescence-associated beta-galactosidase activity was evaluated on 5 μm cryosections of old TNC KO and WT mice inflated with O.C.T. Compound. Representative images of lung sections from old WT **(A,C)** and TNC KO **(B,D)** animals stained for senescence-associated beta-galactosidase activity (blue) and counter-stained with Nuclear Fast Red. *N* = 3 animals/genotype.

To evaluate whether TNC inactivation could affect cellular senescence, if not apoptosis, we checked the senescence-associated beta-galactosidase (SA-β-gal) activity in lung sections of old TNC KO and WT lungs. SA-β-gal activity was essentially observed in epithelial cells of the conducting airways, with no difference between TNC KO and WT samples (Figure 5). However, a distinct punctate staining pattern was also observed throughout the parenchyma, corresponding to alveolar macrophages (or dust cells) phenotype, disseminated throughout the parenchyma. Alveolar macrophages are mononuclear phagocytes recognizable by their large size and foamy cytoplasm, as well as their localization on the internal surface of the alveoli. Alveolar macrophages staining also appeared similar in TNC KO and WT samples.

### Pulmonary Function, However, Remains Equivalent in Old TNC KO and Wild-Type Animals

**FIGURE 6:**
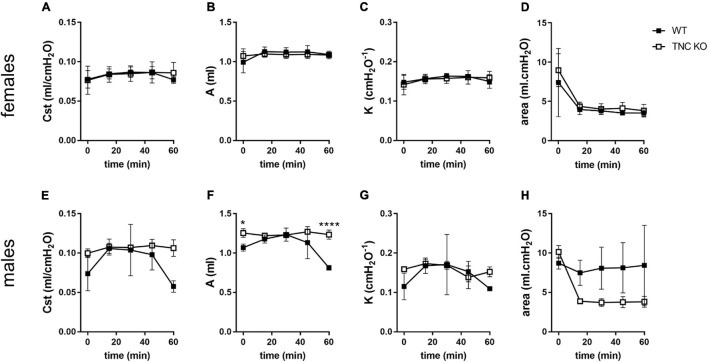
Lung function in old TNC-deficient and WT animals. Respiratory function parameters were collected with the help of a flexiVent system under basal conditions (time 0) or during 1 h of HTV ventilation in old TNC-deficient (□) and WT (■) animals. **(A,E)**: static compliance (Cst), describing the distensibility of the respiratory system; **(B,F)**: parameter A, estimating the inspiratory capacity; **(C,G)**: parameter K, describing the curvature of the PV deflation curve; **(D,H)**: hysteresis, estimating atelectasis existing before the PV loop (area between the PV inflation and deflation limbs). *N* = 3–4 animals/gender and genotype. Results are expressed as mean ± SD. Statistical analyses were made by two-way ANOVA; statistical significance was set at *p* < 0.05; *p < 0.05, ****p < 0.0001.

To identify if larger lung volume, parenchymal volume, total airspace volume and septal surface area were correlated to a modified lung function at old age, we measured respiratory function parameters in anesthetized and mechanically ventilated old TNC-deficient and WT mice. Under basal conditions (time 0 min), static compliance (Cst), an index of the distensibility of the respiratory system, parameter A, an estimate of the inspiratory capacity, parameter K, a measure of the overall curvature of deflation limb of the PV curves, and hysteresis, estimating the atelectasis before the PV loop, were exactly similar in old TNC KO and WT female mice (Figures 6A–D). In males, values tended to be higher in old TNC KO compared to WT animals, but this was only significant for parameter A (Figures 6E–H). As changes in pulmonary function related to age could go unseen in basal conditions, we also evaluated the pulmonary function of old TNC KO and WT male and female mice under a mechanical stress induced by 1 h of a high tidal volume (HTV) profile. Respiratory function parameters were recovered every 15 min. Female TNC KO and WT profiles showed strictly superimposed values for all four parameters tested during all time points of the HTV ventilation (Figures 6A–D). Males results were also similar in TNC KO and WT animals, except that the standard deviations were larger, and time 60 min values were hard to obtain for many of them, indicating a lower level of tolerance to HTV ventilation in males compared to females of both genotypes (Figures 6E–H).

### Non-proliferative Smooth Muscle Cells Population Is Enlarged in Old TNC KO Lungs

**FIGURE 7:**
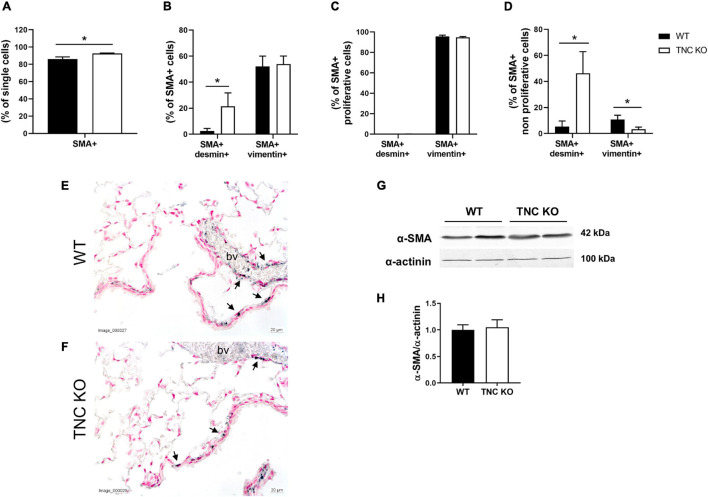
α-SMA positive cells in old TNC-deficient and WT lungs. **(A–D)**: flow cytometry quantification of α-SMA, desmin and vimentin in old female TNC KO and WT lung samples; proliferation status was evaluated using an EdU assay kit. *N* = 3 animals/genotype. Statistical analyses were made by Student’s *t* test; statistical significance was set at *p* < 0.05; *p < 0.05. **(E,F)**: representative images of lung sections from TNC KO and WT old animals immunostained with anti-α-SMA antibody (dark gray; black arrows) and counter-stained with Nuclear Red. *N* = 3–5 animals/genotype. bv: blood vessel. **(G,H)**: representative results of α-SMA protein detection in whole lung lysates from old TNC-deficient and WT animals by Western blot; quantification is shown in histogram; *N* = 6–7 animals/genotype. Cropped blots are displayed.

To determine whether airway smooth muscle (ASM) was concurrently modified in TNC KO lungs, as it was the case in 3 months old TNC-deficient mice, we evaluated α-smooth muscle actin (α-SMA) expression in old female TNC KO and WT lungs by flow cytometry, immunohistochemistry and Western blot (Figure 7). Flow cytometry experiments revealed that the percentage of SMA + cells was very marginally more elevated in old TNC KO lungs compared to their WT counterparts (1.07-fold increase, *p* = 0.01) (Figure 7A). Percentage of SMA + vimentin + cells was similar in both genotypes, but percentage of SMA + desmin + cells was 8 to 9-fold higher in TNC KO than in WT lungs (Figure 7B). Overall proliferation rate of SMA + cells was not significantly affected by TNC inactivation. About half of SMA + cells were proliferating in both genotypes, with unchanged percentage of SMA + vimentin + and SMA + desmin + cells. However, percentage of SMA + vimentin + cells was less (three-fold) and SMA + desmin + cells more (8–9-fold) in the non-proliferative portion of TNC KO SMA + cells, compared to WT counterparts (Figures 7C,D). By immunohistochemistry, we could show that α-SMA staining was found only around small airways and blood vessels, and was comparable in TNC KO and WT lung sections, with fragmented/interrupted staining (Figures 7E,F). Alpha-SMA protein expression evaluated by Western blot in whole lung extracts confirmed an overall conserved expression of the protein in both genotypes (Figures 7G,H).

### TGFβ, TLR4 and Certain Previously Described Integrin-Mediated Molecular Pathways Are Not Up-Regulated in Old TNC KO Lungs

**FIGURE 8:**
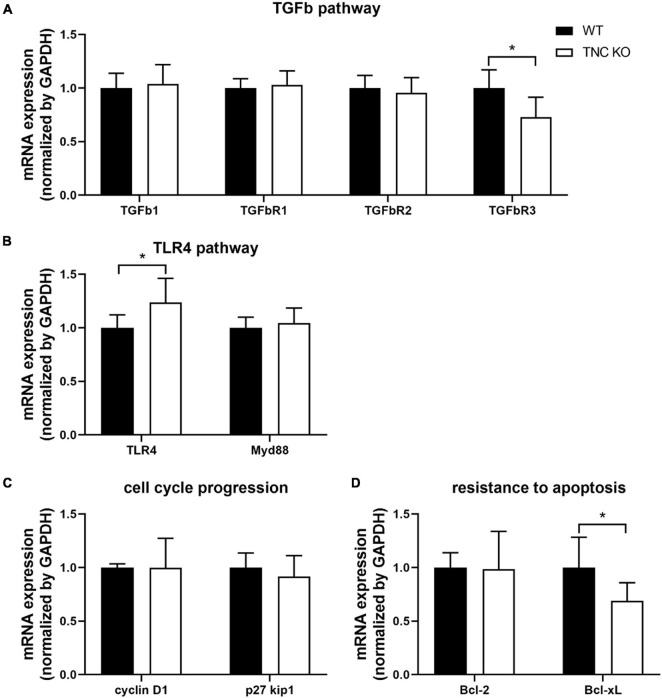
TNC-related molecular pathways in old TNC-deficient and WT lungs. **(A)**: mRNA expression of TGFβ pathway intermediates evaluated by RT-qPCR from whole lung lobe total RNA extractions of old TNC-deficient (□) and WT (■) animals. *N* = 6 animals/genotype. **(B)**: mRNA expression of TLR4 pathway intermediates evaluated by RT-qPCR from whole lung lobe total RNA extraction of old TNC-deficient (□) and WT (■) animals. *N* = 6 animals/genotype. **(C,D)**: mRNA expression of cell cycle proliferation and resistance to apoptosis pathways intermediates, through interaction with integrins, evaluated by RT-qPCR from whole lung lobe total RNA extraction of old TNC-deficient (□) and WT (■) animals. *N* = 6 animals/genotype. Results are expressed as mean ± SD. Statistical analyses were made by Student’s *t* test. Statistical significance was set at *p* < 0.05; *p < 0.05.

We previously showed that mRNA expression of TGFβ and TLR4 pathways intermediates were massively (5–20 times) up-regulated in TNC KO lungs at 3 months of age (Gremlich et al., 2020). In old TNC KO lungs, TGFβ and TLR4 pathways intermediates did not exhibit the same gene over-activation (Figures 8A,B). TGFβ receptor 3 (TGFβR3) mRNA was even slightly down-regulated while TLR4 mRNA was very modestly up-regulated (25% in both cases) in old TNC KO lungs, compared to WT lungs. Two demonstrated downstream signaling pathways linked to integrins activation by TNC were also evaluated. Cell cycle progression markers cyclin D1 and p27kip1 mRNA expressions were unchanged, and anti-apoptotic markers Bcl-2 and Bcl-xL mRNA analysis revealed a 30% decrease in Bcl-xL mRNA expression in TNC KO lungs and no change in Bcl-2 mRNA expression (Figures 8C,D).

## Discussion

Human lung aging is described as a functional decline, with increased residual volume (RV) and functional residual capacity (FRC), reduced regeneration capacity and structurally, airspace enlargement, defined as “senile emphysema” in opposition to the COPD-related inflammatory emphysema. However, human lung evaluations were always fragmentary and better functional and structural analyses of lung aging were provided by animal studies with longitudinal survey of lung morphometry/microstructure and functional parameters in controlled ventilated animals at different ages (Yamamoto et al., 2003; Huang et al., 2007; Elliott et al., 2016; Schulte et al., 2019; Veldhuizen et al., 2019). We aimed at understanding the potential role of TNC in this process.

In the present study, we showed that at 18 months of age, lung volume, parenchymal volume, airspace volume and septal surface area were one-third higher in TNC KO mice, compared to WT counterparts, in males and females. Schulte et al. (2019) recently demonstrated in a very extensive stereological analysis of mouse lungs at 3, 6, 12, 18, and 24 months of age that normal lung aging was accompanied by an increase in lung volume, parenchymal volume, total airspace, ductal airspace and alveolar airspace, occurring between 12 and 18 months. They also showed that septal surface area was concurrently increased (Schulte et al., 2019). Therefore, all the age-related structural modifications described in normal aging in mice were even higher in TNC-deficient mice. These results could either mean that the lung volume is enlarged but the alveoli network is identical (and then the mesh size is larger) (distention of the lung as in lung aging), or that the volume is enlarged but the mesh size is identical (and then the alveoli network is bigger) in TNC KO lungs, compared to their WT counterparts. The length of the free septal edges represents the total length of all alveolar entrance rings. Accordingly, the unchanged length of the free septal edges observed favors the assumption of a conserved mesh size in both genotypes, but in a larger volume. Calculation of a similar Lm in both genotypes strengthened this hypothesis. We previously showed that TNC inactivation led to perturbation of the branching morphogenesis and delays in both phases of alveolarization (classical and continued) during lung development. This was accompanied by a temporary reduction of the total surface area and the total length of the free septal edge, but a catch-up was reached at the age of 3 months, where both TNC KO and WT counterparts had identical lung volume, length of the free septal edge, and septal surface area (Roth-Kleiner et al., 2004; Mund and Schittny, 2020). Consequently, our results implied that somewhere between 3 and 18 months of age, a modification in lung metabolism materialized by structural modifications occurred in TNC KO animals.

An increase in pulmonary volumes and surface area with identical network mesh size could indicate either an enhanced tissue production or a reduced tissue destruction. To figure out the mechanisms underlying these changes, we analyzed proliferation, apoptosis/necrosis and autophagy in the TNC KO and WT lungs, and showed that apoptosis/necrosis markers expression was not altered in old TNC KO lungs, compared to WT lungs (apoptosis was almost undetected in TNC KO and WT lungs). Both autophagy markers LC3bII and p62 proteins were up-regulated in old TNC KO lungs, but as already stated above, activation of autophagy should be related to an increased LC3bII protein but a decreased p62 protein expression. Therefore, autophagy was probably not stimulated in old TNC KO lungs. However, cell proliferation markers PCNA and ki67 were 2 to 3-fold higher in old TNC KO lungs, compared to WT counterparts. Moreover, this was exclusively observed in the parenchymal compartment, which favors the hypothesis of an increased tissue production rather than a decreased cell destruction in old TNC KO lungs, compared to WT lungs. Among the cells present in the parenchyma are alveolar epithelial type I and type II cells (AEC1 and AEC2), immune cells, fibroblasts (and myofibroblasts), and pericytes and endothelial cells forming the capillaries. Proliferation rate and density of AEC1 were previously shown to be decreased in old, compared to young adult mice, contrary to AEC2 (alveolar stem cells and progenitors for AEC1); AEC1 being terminally differentiated cells with no proliferation capacity, the authors concluded that the decrease was probably related to the rate of AEC2 to AEC1 differentiation (Watson et al., 2020). In another study, AEC2 cell density, but not cell number per alveolus, was reported to decrease with age (Schulte et al., 2019). Fibroblast proliferation was also shown to decrease in aged lungs, with a decreased regeneration capacity and a terminal differentiation in myofibroblast (Paxson et al., 2011). Aging is overall linked to a decrease in proliferation capacity, while proliferation was somehow increased or less decreased in old TNC KO lungs, compared to WT counterparts in the present study. Increased stereological measurements observed in normal aging are thought to result more from the loss of supporting tissue rather than a difference in cell proliferation. Therefore, if TNC KO lungs show exaggerated stereological modifications compared to normal aging, they do not come from the same mechanism. It is interesting to note that only short isoforms of TNC mRNA were found to be expressed in adult lungs at all ages tested (3, 6 or 24 months of age) [(Gremlich et al., 2020); data not shown]. Short TNC isoforms are known to promote cell attachment and differentiation, while long isoforms are expressed during organogenesis or in cancer, and promote cell migration and proliferation (Chiquet, 1992; Giblin and Midwood, 2015). The overall increased or less decreased proliferation could then result from the absence of the short TNC isoforms in TNC KO.

Structural modifications of the lung were accompanied by an increase in total collagen content. We had previously shown in 3 months old mice, that TNC inactivation induced a thickening of the collagen layer around small airways compared to WT lungs (Gremlich et al., 2020). In 18 months old lungs, collagen staining around small airways was similar in both genotypes, but total collagen content was doubled, suggesting that the increased collagen was located in the parenchyma, in a diffuse manner. Normal aging is already known to be a condition with increased collagen production and decreased elastin content, although collagen cross-linking or fiber size is also altered (Brandenberger and Muhlfeld, 2017; Sicard et al., 2018). In the present study, collagen content was even higher in the TNC KO lungs, compared to the normal aged WT lungs. Biomechanical properties of lung parenchyma were extensively studied, notably integrating the contribution of collagen and elastin (Suki and Bates, 2011; Suki and Bartolak-Suki, 2015; Bou Jawde et al., 2020). In aging, ECM and parenchymal stiffness were shown to be increased, in part due to an increase in collagen cross-linking occurring with age, and the increased compliance observed in lung aging suggested to result from other modifications like an increase in alveoli size/airspace dimensions. In the present study, we did not find any functional difference between genotypes, despite an increase in collagen content; Lm and total length of the free septal edges values were also similar, although a trend toward higher values in TNC KO lungs, could potentially be perceived (Figures 2G,H).

Senescent cells were shown to accumulate in the lung with aging, which results in increased inflammation, stem cell dysfunction and senescence of adjacent cells; this also favors the development of chronic lung diseases (Campisi, 2016; Brandenberger and Muhlfeld, 2017; Birch et al., 2018; Parikh et al., 2019). In old TNC KO and WT lung samples, senescence-associated β-galactosidase (SA-β-gal) activity was detected in epithelial cells of the conducting airways, and in cells scattered throughout lung parenchyma and morphologically identified as alveolar macrophages. SA-β-gal activity in epithelium of the conducting airways was too variable among samples, to allow any correlation with the genotype. In old mouse lungs, TNC was shown to co-localize with senescent cells, but also to appear ahead of the increase in senescent cells number occurring in aging; moreover, senescent cells were also shown to secrete TNC (Calhoun et al., 2016). It could then be interesting to further characterize senescence appearance in old TNC KO and WT lungs to potentially strengthen the role of TNC in this process suggested in a recent review (Blokland et al., 2020). SA-β-gal staining was also observed in alveolar macrophages in both genotypes in a similar way. However, Hall et al. demonstrated that a proportion of the macrophages expressing SA-β-gal (and p16INK4a, CDK4/6 inhibitor, in their study) were not *bona fide* senescent cells, but a particular class of macrophages with a physiological and reversible polarization. These macrophages retained their phagocytic capability, contrary to *bona fide* senescent cells. They nevertheless accumulated in aged organisms and probably participated in the “inflammaging” process but not through a true senescent phenotype (Hall et al., 2016, 2017). TNC was recently proposed to have a role in innate immune system regulation. It was shown to induce the secretion of several cytokines (IL-6, IL-8 or TNFα) during inflammation or tissue repair, in macrophages, dendritic cells or even fibroblasts, and to promote macrophage migration and influence M1/M2 macrophage polarization according to the context (Shimojo et al., 2015; Sha et al., 2017; Abbadi et al., 2018; Marzeda and Midwood, 2018; Wang et al., 2018; Kimura et al., 2019). In a model of cardiac dysfunction, TNC was even shown to induce inflammation and monocyte/macrophage recruitment, while the effect was abolished in TNC-deficient mice (Abbadi et al., 2018). Therefore, studying more in depth alveolar macrophages in TNC KO and WT mice might be of interest in the context of aging and inflammaging as well.

Mechanistically, TNC can bind to many different types of proteins to activate downstream effectors and mediate its effect on cell proliferation, migration or differentiation: ECM proteins, cell surface receptors or soluble proteins such as proteases or growth factors for example (Humphries et al., 2006; Midwood and Orend, 2009; Midwood et al., 2016). Transforming growth factor β (TGFβ) was shown to be one of the major modulator of TNC expression, and Toll-like receptor 4 (TLR4) and integrins, major cell surface receptors binding TNC [reviewed in (Chiovaro et al., 2015; Tucker and Chiquet-Ehrismann, 2015; Yoshida et al., 2015; Bhattacharyya et al., 2016; Midwood et al., 2016; Marzeda and Midwood, 2018; Yalcin et al., 2020)]. We had previously shown that several TGFβ and TLR4 pathway intermediates were tremendously up-regulated at the mRNA level in 3 months old TNC KO lungs, compared to WT lungs (5–20-times fold increase) (Gremlich et al., 2020). In the present study, mRNA expression of TGFβ and TLR4 pathway intermediates were roughly similar in lungs of both genotypes, meaning that TNC inactivation effect on cell proliferation was not going through an over-activation of these pathways. However, TGFβR3 mRNA expression was slightly decreased and TLR4 mRNA slightly increased. TGFβR3 is a co-receptor, which binds to TGFβR ligands to regulate their interaction with TGFβR1/2. In the lung, during TGFβ-induced myofibroblast differentiation, TGFβR3 expression was shown to be inhibited concomitantly with the increase in α-SMA and pro-collagen type I expression; conversely, if TFβR3 was reintroduced, differentiation was inhibited (Ahn et al., 2010). Knowing that TNC induces myofibroblast differentiation, it is surprising that the only TGFβ superfamily member with a modified mRNA expression in TNC KO lungs is TGFβR3, and that its negative regulation rather favors myofibroblast differentiation. However, total myofibroblast population was overall not affected by TNC inactivation in the present study, as shown by the flow cytometry experiment. As for TLR4 mRNA expression, the small increase detected in TNC KO lungs could possibly be an attempt of compensation for TNC inactivation the same way as in 3 months old lungs (Gremlich et al., 2020). Molecular pathways by which TNC was able to enhance cell proliferation through integrins binding in pancreatic cancer cells have recently been described in two publications. Cai et al. (2018) showed that TNC controlled cell cycle progression by acting on the AKT/FOXO1 pathway, resulting in p27kip1 gene (a cyclin-dependent kinase inhibitor) down-regulation and cyclin D1 gene up-regulation. Both effects allow the cells to pass the G1/S restriction point and enter the S phase of DNA replication, by promoting cyclin D-CDK4/6 complex formation (Suryadinata et al., 2010). Shi et al. (2015) also showed that TNC promoted apoptosis resistance through the ERK/NF-kB pathway, which ended in anti-apoptotic Bcl-2 and Bcl-xL gene expression stimulation. In the present study, neither cyclin D1 nor p27kip1 mRNA expression were altered in old TNC KO lungs, compared to WT counterparts. However, mRNA expression of Bcl-xL, but not Bcl-2, was reduced in TNC KO lungs, in accordance with the literature. This could indicate that TNC inactivation might additionally modulate cell proliferation through an increase in apoptosis in old TNC KO lungs. However, we could almost not detect any apoptosis in 18 months old TNC KO or WT lungs.

Lung function decline with age has been well described in mice, and is characterized by an increase in static compliance (Cst) and inspiratory capacity (IC) (Elliott et al., 2016; Kling et al., 2017; Schulte et al., 2019; Veldhuizen et al., 2019). In the present study, old TNC-deficient mice did not show any difference in basal Cst or IC, compared to their WT counterparts, even if males tended to have higher basal values in TNC-deficient mice than in WT mice (only significant for IC). As for HTV ventilation, female and male old TNC KO and WT mice showed superimposed curves, at least during the first 45 min for the males. Indeed, old males were less resistant to mechanical ventilation than females, showed greater standard deviations between individuals, and had functional parameters values that tended to fall after 45 min of HTV ventilation (especially in WT animals). Surprisingly, old TNC KO and WT mice had similar basal Cst and IC values as TNC KO mice at 3 months of age. Indeed, we previously showed that young adult TNC KO mice had higher Cst and IC values than their WT counterparts (Gremlich et al., 2020; Supplementary Figure 3). This means that 3 months old TNC KO mice already exhibited the Cst and IC observed in normal aging, as a premature lung aging phenotype, knowing that these functional changes were described to occur between 3 and 6 months of age in a very extensive study on mouse lung aging (Schulte et al., 2019).

Using flow cytometry, we showed that the percentage of α-SMA + cells was very marginally increased in 18 months old TNC KO lungs compared to WT counterparts. Quantitative analysis of α-SMA protein expression in whole lung protein extracts also resulted in identical expression of the protein in old TNC KO and WT lungs. Qualitative analysis of α-SMA by immunohistochemistry showed that α-SMA staining appeared similar in old TNC KO and WT lung sections, with a fragmented staining around bronchioles and blood vessels, but no staining in the parenchyma, as was already the case in young adult mice at 3 months of age (Gremlich et al., 2020). Flow cytometry analyses, however, revealed that the percentage of SMA + desmin + cells was very much higher in old TNC KO lungs than in WT lungs, and that these cells were essentially non-proliferative cells. Non-proliferative SMA + vimentin + cells were conversely decreased in TNC KO lungs, compared to WT lungs, but the major part of SMA + vimentin + cells were proliferative cells, present in the same proportion in both genotypes. Myofibroblasts are normally not present in adults, but appear *de novo* during repair processes or morphogenesis by differentiation from several types of cells, like fibroblasts, pericytes or even mesenchymal progenitor cells (MOC) (Phan, 2008, 2012). With age, lung myofibroblasts were shown to gradually lose their de-differentiation capacity, adopt a senescent phenotype and become resistant to apoptosis (Paxson et al., 2011; Yanai et al., 2015; Kato et al., 2020). TNC promotes myofibroblast differentiation by inducing SMA and collagen type I mRNA expression, but TNC mRNA expression is also induced during myofibroblast differentiation through TGFβ pathway (Imanaka-Yoshida et al., 2001; Tamaoki et al., 2005; Pegorier et al., 2010; Marzeda and Midwood, 2018; Katoh et al., 2020). Therefore, TNC inactivation could be in part responsible for the lower proportion of myofibroblasts in the non-proliferative pool of SMA + cells. As for SMA + desmin + cells, a general thickening of vascular walls was described in aging, due to VSMC proliferation, in addition to perturbation in elastin and collagen deposition (Xu et al., 2017). VSMC were shown to shift from a contractile phenotype (differentiated cells) to a synthetic phenotype (dedifferentiated cells) with age, with increased migration from the tunica media into the tunica intima of blood vessels, increased proliferation and increased ECM synthesis (Monk and George, 2015; Lacolley et al., 2018; Sicard et al., 2018). With respect to airway wall thickening, it was previously thought to occur with a higher frequency in old subjects; however, a more recent report using high-resolution computed tomography could show that airway wall thickness was rather decreased between 20 and 80 years, with a good correlation factor (Telenga et al., 2017). Our flow cytometry experiment did not allow us to distinguish between VSMC and ASM cells. However, an increased VSMC population in TNC KO lungs would indicate an exaggeration of the vascular wall thickening already observed in normal aging. Further studies looking more precisely at the blood vessels in the distal lung might maybe shed some light on the contribution of VSMC to the increased SMA + desmin + population cited above.

**FIGURE 9:**
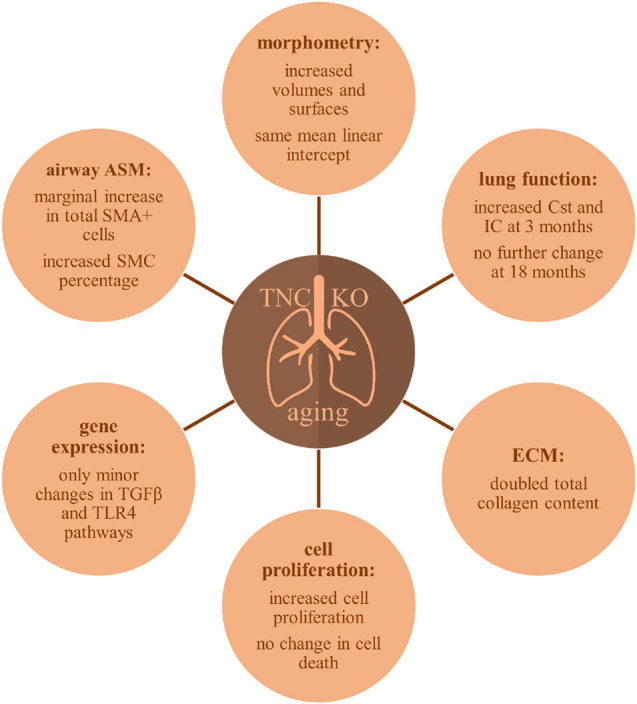
Schematic overview of TNC inactivation effects in aging lung.

A schematic representation of TNC inactivation effects in lung aging is presented in Figure 9. Old TNC KO mice showed morphometric results compatible with exaggerated features of lung aging, such as increased lung volume, parenchymal volume, total airspace volume and septal surface compared to their WT counterparts, already shown to have increased volume and surface values compared to young mice (Schulte et al., 2019). This was associated with an increased collagen content in TNC KO lungs compared to WT lungs, a feature already existing in normal aging. Analysis of PV curves showed that basal Cst and IC values were identical in old TNC KO and WT mice, but were identical to 3 months old TNC KO mice values, themselves higher than their WT counterparts (Gremlich et al., 2020). Increased Cst and IC were previously shown to be changes occurring with normal lung aging between 3 and 6 months of age, while structural alterations essentially occurred between 12 and 18 months of age (Schulte et al., 2019). Collectively, several differences observed were compatible with exaggerated features of lung aging in TNC-deficient animals. As a weak expression of TNC was consistently reported in adult lung (Koukoulis et al., 1991; Natali et al., 1991; Kaarteenaho-Wiik et al., 2001; Liesker et al., 2009; Lofdahl et al., 2011), these effects might either still be inherited from the developmental alterations observed in fetal or neonatal life (Roth-Kleiner et al., 2004, 2014; Mund and Schittny, 2020), or result from the basal low TNC expression remaining at all ages. Further experiments on TNC might anyhow show an improvement of lung aging with TNC supplementation.

## Data Availability Statement

The original contributions presented in the study are included in the article/Supplementary Material, further inquiries can be directed to the corresponding author.

## Ethics Statement

The animal study was reviewed and approved by the Animal Ethics Committee of the Federal Food Safety and Veterinary Office, and the Veterinary Service of the Canton Bern.

## Author Contributions

EY and JS: breeding and handling of the mice. EY, JS, SG, and TC: harvesting of the lungs. EY: embedding of the tissue and histological stainings. SG, TC, and JS: morphological observations. SG: immunohistochemistry, western blots, senescence assay, apoptosis assay, RT-qPCR, and writing the first draft. TC and SG: lung function tests. KF: flow cytometry. FC: stereology. JS, MR-K, and SG: conceive the study. All authors read and approved the manuscript.

## Conflict of Interest

The authors declare that the research was conducted in the absence of any commercial or financial relationships that could be construed as a potential conflict of interest.

## Publisher’s Note

All claims expressed in this article are solely those of the authors and do not necessarily represent those of their affiliated organizations, or those of the publisher, the editors and the reviewers. Any product that may be evaluated in this article, or claim that may be made by its manufacturer, is not guaranteed or endorsed by the publisher.
