# Supplementary material for: Tenascin-C: Friend or Foe in Lung Aging?
Source: Front Physiol. 2021 Oct 27;12:749776. doi: 10.3389/fphys.2021.749776 (PMC8578707; doi:10.3389/fphys.2021.749776)
Supplement: Supplementary file 1 [file Table_1.pdf]

Supplementary Table I: Primers panel for RT-qPCR.

	**forward**	**reverse**
**TGFβ1**	5’-CTG CTG ACC CCC ACT GAT AC-3’	5’-AGC CCT GTA TTC CGT CTC CT-3’
**TGFβR1**	5’-GCA TTG GCA AAG GTC GGT TT-3’	5’-TGC CTC TCG GAA CCA TGA AC-3’
**TGFβR2**	5’-GTG AGA CTG TCC ACT TGC GA-3’	5’-TGT CGT TCT TCC TCC ACA CG-3’
**TGFβR3**	5’-CTG CCA AGG GAG GTT CAC AT-3’	5’-AGC AGG AAC ACA ACA GGC TT-3’
**TLR4**	5’-AGC CGG AAG GTT ATT GTG GT-3’	5’-CAG CAG GGA CTT CTC AAC CT-3’
**MyD88**	5’-CCG CCT ATC GCT GTT CTT GA-3’	5’-CCA GGC ATC CAA CAA ACT GC-3’
**ki67**	5’-ATT GAC CGC TCC TTT AGG TAT GAA-3’	5’-TTG ACC TTC CCC ATC AGG GTC-3’
**PCNA**	5’-TCG TCT CAC GTC TCC TTG GT-3’	5’-TTT TGG ACA TGC TGG TGA GGT-3’
**cyclin D1**	5’-CAA AAT GCC AGA GGC GGA TG-3’	5’-CAT GGA GGG TGG GTT GGA AA-3’
**p27kip1**	5’-AGT TCT ACT ACA GGC CCC CG-3’	5’-CCA ATT AAA GGC ACC GCC TG-3’
**Bcl-2**	5’-GAC AAC ATC GCC CTG TGG AT-3’	5’-AGC CCA GAC TCA TTC AAC CA-3’
**Bcl-xL**	5’-CGG ATT GCA AGT TGG ATG GC-3’	5’-TGC TGC ATT GTT CCC GTA GA-3’
