# Supplementary material for: Tenascin-C: Friend or Foe in Lung Aging?
Source: Front Physiol. 2021 Oct 27;12:749776. doi: 10.3389/fphys.2021.749776 (PMC8578707; doi:10.3389/fphys.2021.749776)
Supplement: Supplementary file 2 [file Image_1.pdf]

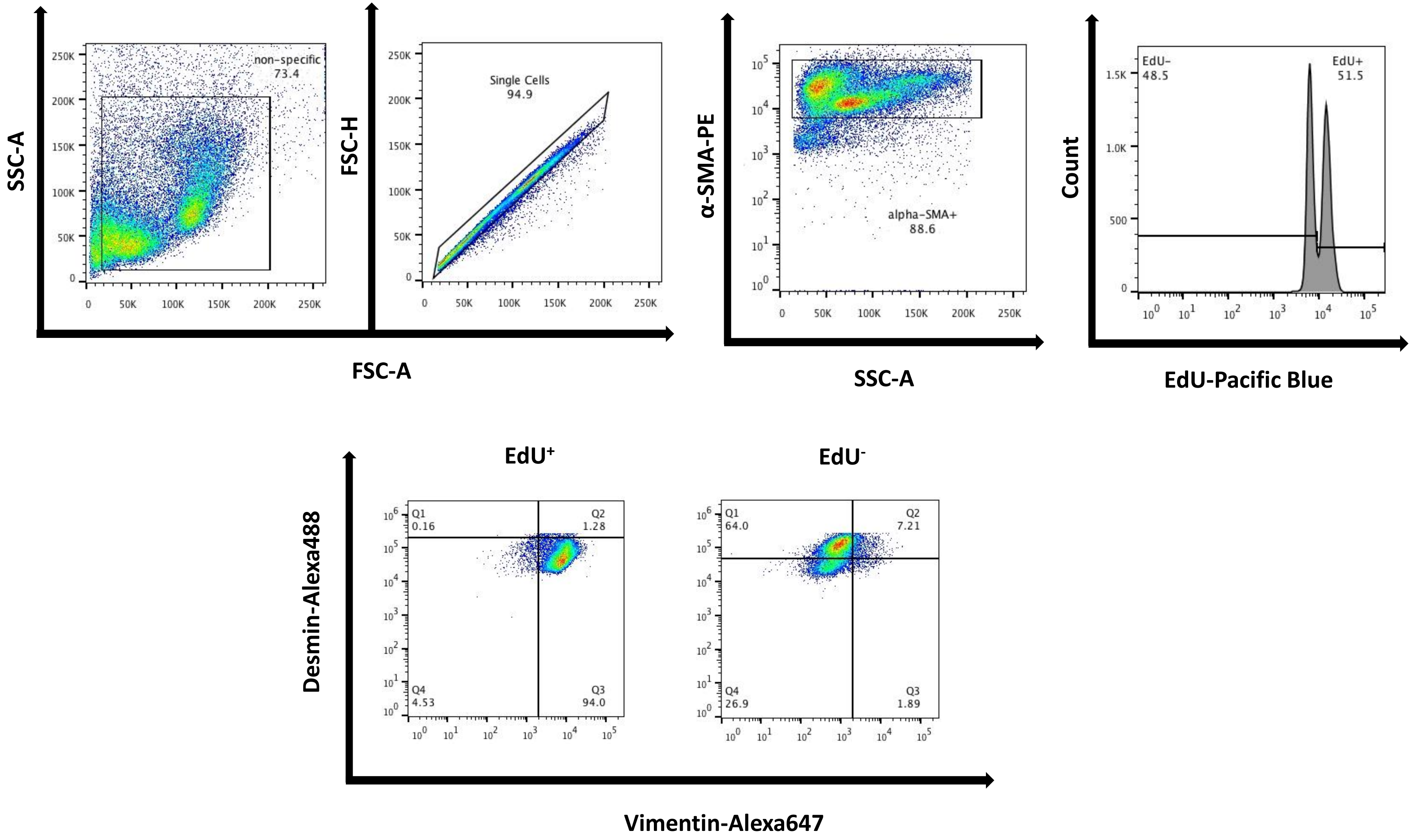

Supplementary Figure 1: Flow cytometry gating strategy. A representative gating strategy of the cellular characterization from mouse lung homogenates is depicted. Initially, dead cells were gated out *via* size exclusion (FSC-A *vs* SSC-A) and single cells were selected (FSC-A *vs* FSC-H). Cells that express α-SMA (α-SMA vs SSC-A) were selected and proliferation levels were measured. The two distinct peaks (EdU-Pacific Blue) discriminates proliferative cells (EdU^+) from non-proliferative cells (EdU^-). Then the two distinct populations were analyzed for Myofibroblasts (α-SMA^+/$Vimentin^+$/$Desmin^-$) and SM cells (α-SMA^+/$Vimentin^-$/$Desmin^+$).
