# Supplementary material for: Tenascin-C: Friend or Foe in Lung Aging?
Source: Front Physiol. 2021 Oct 27;12:749776. doi: 10.3389/fphys.2021.749776 (PMC8578707; doi:10.3389/fphys.2021.749776)
Supplement: Supplementary file 3 [file Image_2.pdf]

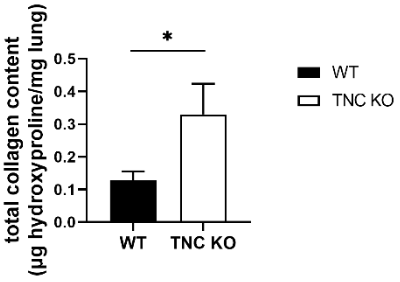

Supplementary Figure 2: Total collagen content was doubled in TNC KO lungs. Total collagen content was measured by the hydroxyproline assay, starting from frozen lungs. N=3 animals/genotype. Results are expressed as mean ± SD. Statistical analysis was made by Student's t test; statistical significance was set at $p<0.05$; * $p<0.05$.
