# Supplementary material for: Tenascin-C: Friend or Foe in Lung Aging?
Source: Front Physiol. 2021 Oct 27;12:749776. doi: 10.3389/fphys.2021.749776 (PMC8578707; doi:10.3389/fphys.2021.749776)
Supplement: Supplementary file 4 [file Image_3.pdf]

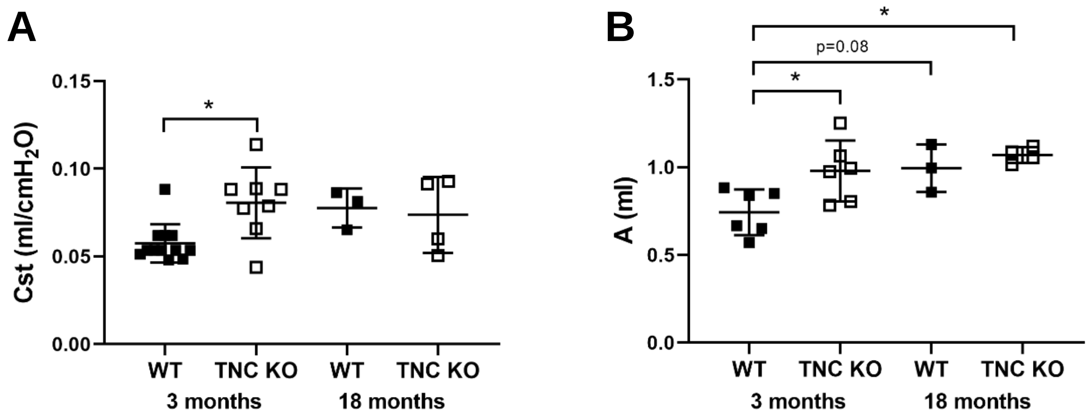

Supplementary Figure 3: 18 months old TNC KO and WT mice have similar basal static compliance (Cst) and inspiratory capacity (A) values as 3 months old TNC KO mice. Respiratory function parameters were collected with the help of a flexiVent system under basal conditions in TNC-deficient (□) and WT (■) animals at 3 and 18 months of age. (A): static compliance (Cst), describing the distensibility of the respiratory system; (B): parameter A, estimating the inspiratory capacity. N=6-12 animals/genotype for 3 months old animals and 3-4 for 18 months old animals. Results are expressed as mean ± SD. Statistical analyses were made by two-way ANOVA; statistical significance was set at $p<0.05$; * $p<0.05$.
